# Special Delivery: Potential Mechanisms of Botulinum Neurotoxin Uptake and Trafficking within Motor Nerve Terminals

**DOI:** 10.3390/ijms21228715

**Published:** 2020-11-18

**Authors:** Brittany M. Winner, Skylar M. L. Bodt, Patrick M. McNutt

**Affiliations:** 1 United States Army Medical Research Institute of Chemical Defense, Gunpowder, MD 21047, USA; winnerbr@msu.edu; 2 Department of Cellular and Molecular Physiology, Penn State College of Medicine, Hershey, PA 17033, USA; smb846@psu.edu; 3 Wake Forest Institute for Regenerative Medicine, Wake Forest University, Winston-Salem, NC 27101, USA

**Keywords:** toxins, botulinum neurotoxin, synaptic endocytosis, bulk endosome, translocation, synaptic vesicles

## Abstract

Botulinum neurotoxins (BoNTs) are highly potent, neuroparalytic protein toxins that block the release of acetylcholine from motor neurons and autonomic synapses. The unparalleled toxicity of BoNTs results from the highly specific and localized cleavage of presynaptic proteins required for nerve transmission. Currently, the only pharmacotherapy for botulism is prophylaxis with antitoxin, which becomes progressively less effective as symptoms develop. Treatment for symptomatic botulism is limited to supportive care and artificial ventilation until respiratory function spontaneously recovers, which can take weeks or longer. Mechanistic insights into intracellular toxin behavior have progressed significantly since it was shown that toxins exploit synaptic endocytosis for entry into the nerve terminal, but fundamental questions about host-toxin interactions remain unanswered. Chief among these are mechanisms by which BoNT is internalized into neurons and trafficked to sites of molecular toxicity. Elucidating how receptor-bound toxin is internalized and conditions under which the toxin light chain engages with target SNARE proteins is critical for understanding the dynamics of intoxication and identifying novel therapeutics. Here, we discuss the implications of newly discovered modes of synaptic vesicle recycling on BoNT uptake and intraneuronal trafficking.

## 1. Introduction

**Figure 1:**
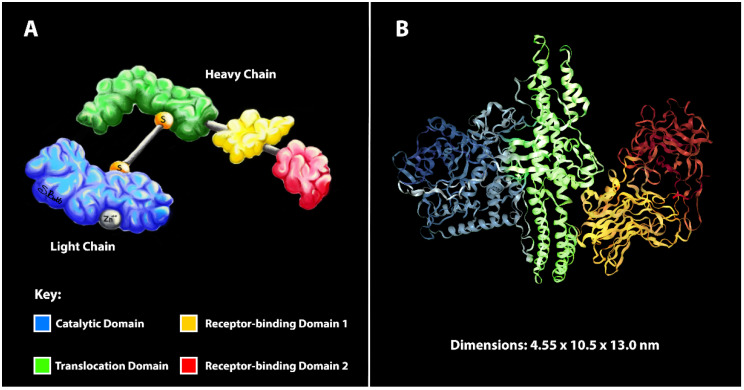
Structure-function relationships of botulinum neurotoxins (BoNTs). (**A**) Cartoon summarizing the various domains of BoNT holotoxin. Functional domains are distinguished by colors according to the key. The catalytic zinc is grey and the disulfide bond is formed between two cysteines (orange). (**B**) Ribbon structure of BoNT/A, including unit dimensions of the crystallized toxin from [4]. Colors correspond to panel (**A**).

**Figure 2:**
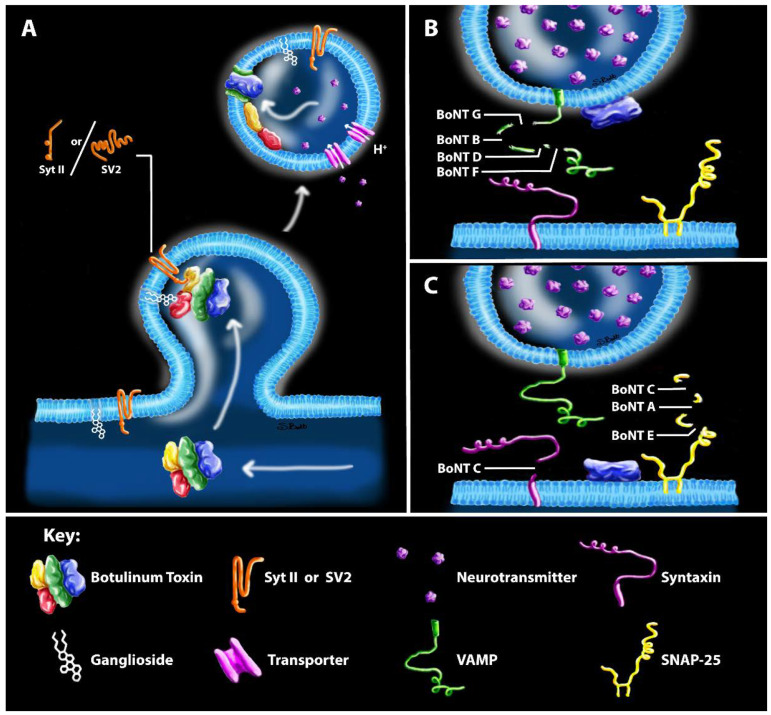
Classical model of toxin uptake and catalytic engagement with SNARE proteins. (**A**) Toxin uptake occurs through binding of a protein and lipid co-receptors and internalization via synaptic endocytosis. (**B**) Summary of LCs that cleave VAMP. (**C**) Summary of LCs that cleave SNAP-25. Structures correspond to the key at the bottom of the figure.

Botulinum neurotoxins (BoNTs) comprise a family of closely related bacterial protein toxins that are serologically grouped into serotypes, many of which contain multiple subtypes (e.g., A1–A8) [1]. BoNTs are synthesized as single chain proteins, which are proteolytically activated to mature heterodimers consisting of a light chain (LC; ~50 kDa) and a heavy chain (HC; ~100 kDa) that remain associated via a peptide belt, non-covalent interactions and an interchain disulfide bond (Figure 1A,B) [2]. The two domains have distinct functions: HC mediates the precise recognition of neuron-specific receptors on the presynaptic membrane, facilitates neuronal uptake via synaptic endocytosis and mediates translocation of LC through the endosomal membrane into the presynaptic cytosol (Figure 2A), whereas LC is a Zn^2+^-dependent metalloprotease which specifically cleaves unique dipeptide targets in vesicle-associated membrane protein 1-3 (VAMP1-3; serotypes B, D, F, G; Figure 2B), synaptosomal-associated protein-25 (SNAP-25; Figure 2C), or syntaxin-1 (STX-1; Figure 2C). Cleavage of SNAP-25, VAMP1-3 and STX-1 by LC prevents synaptic vesicle (SV) fusion and neurotransmitter release, causing the flaccid paralysis and respiratory depression characteristic of clinical botulism (Figure 2B,C) [3]. 

The intracellular molecular toxicity of BoNT results from a carefully orchestrated series of interactions with neuronal proteins. Intoxication involves three major events, which occur in distinct physiological compartments: (1) binding of HC to neuron-specific receptors exposed on presynaptic membranes, enabling neuronal uptake via synaptic endocytosis (Figure 2A); (2) translocation of LC into the cytosol through a pore formed by HC in response to endosome acidification (Figure 2A); and (3) LC proteolysis of presynaptic SNARE proteins (Figure 2B,C) [5]. The deadly combination of neuronal specificity and the precise cleavage of neuronal SNARE proteins in the presynaptic compartment render BoNTs the most potent poisons known, with estimated human LD_50_ values as low as 0.1–1 ng/kg [5].

The primary clinically relevant target of BoNT is the peripheral cholinergic nerve terminal. Clinical symptoms of botulism manifest within 12–36 h as cranial palsies that can rapidly progress to skeletal muscle weakness and death from respiratory failure [6]. To date, there is no antidote for botulism. Post-exposure prophylaxis with neutralizing equine antitoxin is effective in preventing symptomatic onset [7,8]. However, antitoxins have no effect on BoNT that has entered the presynaptic compartment, and thus have marginal therapeutic value in symptomatic patients. In the absence of an antidotal treatment, intubated patients must be maintained on artificial ventilation until the spontaneous recovery of respiratory function, which typically takes weeks [9,10]. As illustrated during the recent coronavirus epidemic [11], intensive care facilities have limited capacity to accommodate a surge of patients, and thus even small outbreaks of botulism can strain or exhaust the medical resources of major metropolitan areas [12,13]. Despite the urgent need for post-symptomatic treatments, clinically effective small molecule inhibitors that inhibit intracellular LC proteolytic activity and exhibit acceptable pharmacokinetic parameters have not been identified for any BoNT serotype. Although extensive understanding is now available regarding structural and functional aspects of substrate binding and proteolysis, selective inhibition of LC proteolytic activity within the cellular environment has proven difficult [1].

While many aspects of the molecular interactions involved in BoNT toxicity have been described, including receptor binding [14], translocation [15,16], and proteolytic mechanisms [17,18], toxin interactions with neuronal proteins involved in intracellular trafficking and molecular toxicity remain poorly understood. In principle, the development of therapeutics that disrupt conserved aspects of intraneuronal toxin interactions, such as translocation inhibitors [19], have broad-spectrum potential against the extensive functional diversity exhibited by the different BoNT subtypes. However, characterizing these conserved interactions is challenging, in part because the extraordinarily small amounts of toxin that are sufficient to paralyze neuromuscular junctions (estimated to be less than 100 molecules) limit the use of conventional molecular approaches to characterize intracellular toxin behavior under physiological conditions [20]. Here, we review recent findings regarding mechanisms of synaptic endocytosis and SV regeneration, with the goal of illuminating poorly understood aspects of toxin trafficking within the presynaptic compartment.

## 2. Does the Specific Mechanism of Toxin Internalization into Neurons Affect Toxin Uptake?

Following SV fusion with presynaptic membranes, excess membrane and vesicular proteins are organized and retrieved through a choreographed process known as synaptic endocytosis [21]. BoNT exploits this process by engaging in high-affinity molecular interactions with lumenal domains of SV proteins SV2 (serotypes A and E) or SYT1/2 (serotypes B, D, F and G) presented from the presynaptic membrane after vesicle fusion, thus promoting neuron-specific uptake [22,23]. Recent studies reveal synaptic vesicle proteins are retrieved from presynaptic membranes through multiple modes of endocytosis, leading to the intriguing hypothesis that BoNT uptake and toxicokinetic progression is influenced by the mode of endocytosis

### 2.1. Proposed Routes of Toxin Uptake by Synaptic Endocytosis

**Figure 3:**
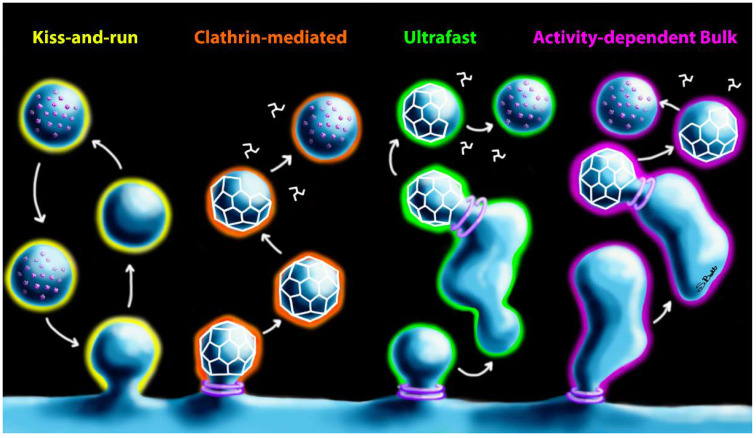
Comparison of the four proposed modes of synaptic endocytosis: kiss-and-run, clathrin-mediated endocytosis (CME), ultrafast endocytosis (UFE), and activity-dependent bulk endocytosis (ADBE). Formation of the clathrin coat is represented by white cages, with clathrin disassembly depicted by white pinwheels. Purple rings represent dynamin rings during membrane scission. Purple spheres indicate neurotransmitter filling during SV biogenesis. For all modes of endocytosis, the final stage is a regenerated SV ready for inclusion in release pools.

Ultrastructural imagery, electrical capacitance measurements, and real-time imaging of fluorescently tagged SV molecules have identified four functionally distinct forms of compensatory endocytosis: clathrin-mediated endocytosis (CME); ultrafast endocytosis (UFE); activity-dependent bulk endocytosis (ADBE); and kiss-and-run endocytosis (Figure 3) [24,25,26,27]. CME, which is the classical model of synaptic endocytosis, involves clathrin coating and dynamin-mediated fission of nascent SVs directly from peri-active zone membrane [28]. Clathrin acts as a molecular sorter, organizing protein content to ensure nascent SVs have the molecular composition necessary for vesicle fusion [29,30]. Following clathrin uncoating, vesicular ATPase acidifies the lumen and initiates neurotransmitter filling [31] and SVs rejoin presynaptic release pools approximately 10–100 s after endocytosis. Consistent with the slow pace, CME occurs in response to low-frequency nerve stimulation, when release pools are not stressed by exhaustion [32,33,34,35]. Notably, the addition of dynamin inhibitors reduces SNARE protein cleavage in cultured neurons exposed to BoNT, which was interpreted to indicate that BoNT uptake is mediated by CME [36,37]. However, recent studies indicate that clathrin also mediates SV biogenesis from synaptic endosomes under physiological conditions [38,39], including endosomes formed during ADBE and UFE [40]. Consequently, the antagonism of BoNT intoxication by dynamin inhibitors is consistent with toxin uptake by multiple forms of synaptic endocytosis. 

UFE is predominantly observed under conditions of mild stimulation intensity, although UFE may also contribute to compensatory endocytosis during high-frequency stimulation [41,42]. UFE retrieves endosomes that are approximately 80 nm in diameter within 50–100 ms after SV fusion from peri-active zone membrane in an actin-dependent, clathrin-independent manner [39,41,42,43]. Following internalization, UFE vesicles fuse with extant synaptic endosomes for protein sorting and clathrin-dependent SV biogenesis, regenerating SVs within 3–5 s after fusion events [39].

At higher stimulation frequencies, UFE and CME cannot compensate for the quantity of SV material deposited on the active zone membrane [44]. Under these conditions, the high-capacity recovery of SV material occurs through actin-dependent, clathrin-independent endocytosis of large vesicles (150–500 nm) in a process known as ADBE [32,43,45,46]. ADBE vesicles develop directly into synaptic endosome-like structures which produce new SVs via clathrin-mediated biogenesis, with a time frame of 0.5–1 min [35,39]. A principal difference between UFE and ABDE is that the ABDE involves large-scale retrieval of membrane that directly transitions to synaptic endosome-like structures, whereas UFE endosomes merge with pre-existing synaptic endosomes. ADBE is triggered by physiological stimuli in neuromuscular preparations [46], suggesting that ABDE may play a dominant role in BoNT endocytosis.

Kiss-and-run endocytosis results from a reversible hemifusion event, in which an SV partially merges with the presynaptic membrane then rapidly disengages [47]. Similar to CME, kiss-and-run maintains vesicular identity regarding membrane and protein composition, circumventing the need for resorting of SV cargo and allowing for the regeneration of SVs within 0.5 s [48,49,50,51]. The kiss-and-run pore is estimated to be 1–5 nm in diameter [52,53] and has a short duration of existence, resulting in the limited exchange of SV cargo compared to a full fusion event [54]. Furthermore, BoNT/A dimensions are approximately 4.55 × 10.5 × 13.0 nm, suggesting that the toxin is too large to negotiate the fusion pore during the transient fusion event [4]. Current evidence argues against a major role for kiss-and-run endocytosis in mammalian neurons under physiological conditions [25,27]. Since kiss-and-run endocytosis appears to be unlikely to meaningfully contribute to BoNT intoxication, it is not discussed further.

### 2.2. Functional Implications of Endocytosis Mode on Intracellular Toxin Activity

LC translocation from the endosome to the cytosol is initiated by endosome acidification and culminates with thioredoxin reductase-mediated reduction of the interchain disulfide bond, freeing the LC on the cytosolic side of the endosomal membrane [19,55]. As described above, endosome acidification occurs at different stages depending on the specific mode of synaptic endocytosis [56]. For example, while acidification occurs in individual SVs during CME, acidification occurs in bulk endosomes following UFE and ABDE [57,58]. The specific details of vesicle acidification are of particular interest given that LC translocation is required for molecular toxicity [19]. It is presumed that BoNT light chains targeting SNAP-25 (sLCs) exert their toxic effect at the presynaptic membrane, whereas LCs targeting VAMP1-3 (vLCs) exert their toxic effect on individual SVs. However, the stage of SV recycling at which sLC and vLC engage their respective molecular targets has not been experimentally determined. Here, we discuss two possible models explaining LC molecular toxicity: the classical model, in which LC translocation occurs after SV biogenesis; and a new model, in which LC translocation occurs at the bulk endosome stage prior to SV biogenesis. In both models, we consider the hypothesis that translocated LC remains directly or indirectly associated with endosomal or vesicle membranes, following movement though the HC pore and reduction of the interchain disulfide bond. According to this hypothesis, LC is constrained to a two-dimensional surface and trafficked to be in close proximity to its target substrates, thus contributing to the high potency of BoNT. Alternatively, if the toxin is released to diffuse within the cytosol after translocation, the light chain would be free to engage membrane-associated SNARE proteins throughout the synaptic volume; however, the tortuous presynaptic architecture and comparatively low toxin-substrate density may constrain diffusion and offer reduced opportunity for toxin-substrate interaction [59].

**Figure 4:**
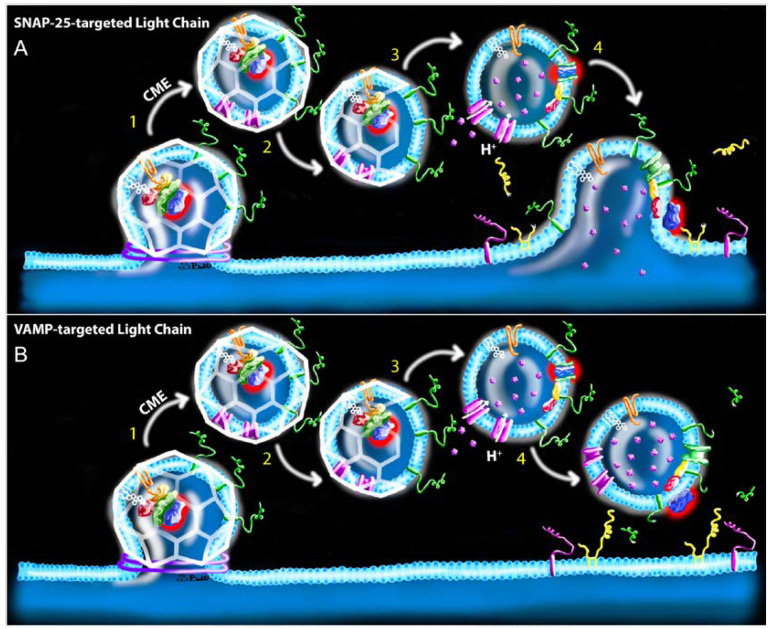
Hypothesized mechanisms of toxin uptake and LC activity following CME. (**A**) VAMP-targeted LC. Following dynamin-dependent, clathrin-coated endocytosis (step 1), the clathrin coat (white) is disassembled (step 2) and the vATPase proton pump (pink) acidifies the endosome, allowing LC (blue with red halo) translocation to the cytosolic membrane surface (step 3). Translocated vLC cleaves VAMP proteins (green), producing fusion-incompetent SVs that are able to dock and futilely occupy release sites (step 4). (**B**) SNAP-25-targeted LC. Steps 1–3 are as identical as the top panel. However, in contrast to vLC-associated SVs, sLC-associated SVs remain fusion-competent following translocation in step 4, thus releasing sLC onto the presynaptic membrane where it cleaves SNAP-25 proteins (yellow). Structures correspond to the key in Figure 2.

During CME, vesicle acidification occurs after disassembly of the clathrin coat [31]. Regenerated CME vesicles then move directly to SV release pools. There is no evidence that LC can move among SVs without a fusion event; indeed, translocated LC/A appears to remain associated with lipid membrane [60]. The hypothesis that translocated LC remains associated with the same CME-derived SV until membrane fusion has interesting serotype-specific implications for LC trafficking and the engagement of target SNARE proteins. For example, sLC targets SNAP-25, which is primarily located on the presynaptic membrane [61,62]. Consequently, sLC-associated SVs must undergo an additional round of fusion for sLC to engage with SNAP-25 on the presynaptic membrane (Figure 4A). In comparison, vLC could cleave VAMP directly on SVs (Figure 4B), without requiring additional fusion events. Once release sites are associated with cleaved SNAP-25 or SVs containing cleaved VAMP, SV will dock but be unable to undergo fusion, thus having a dominant-negative effect on release until the SV spontaneously disengaged from the release site [63,64,65].

**Figure 5:**
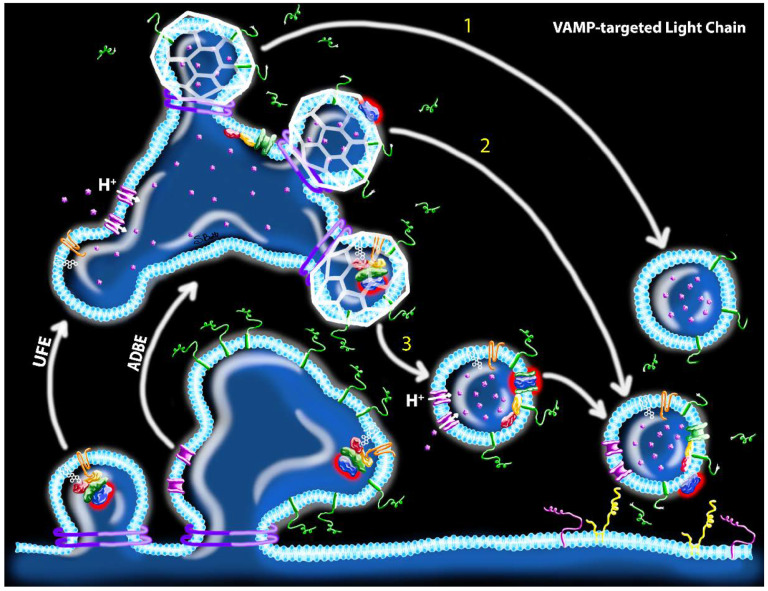
Hypothesized mechanisms of toxin uptake and potential proteolytic sites of action of VAMP-targeted LCs following UFE or ABDE. After fission from the presynaptic membrane, ABDE produces a bulk endosome, whereas UFE produces an endosome vesicle that subsequently fuses with a bulk endosome. vATPase proton pumps (pink) are activated during the bulk endosome stage, leading to slow acidification of bulk endosomes. Nascent SVs are produced from bulk endosomes via clathrin-coated (white) biogenesis with three potential outcomes: (1) vLC translocation (blue with red halo) and VAMP (green) cleavage occurs at the bulk endosome stage, producing SVs with cleaved VAMP that do not carry a vLC; (2) Similar to step 1, except a translocated vLC is captured on the SV during biogenesis; and (3) Acidification of the bulk endosome is not sufficient to drive translocation and intact holotoxin is captured within the SV during biogenesis. vLC translocation occurs after clathrin uncaging, producing SVs with active vLC that subsequently cleaves VAMP. In all three scenarios, the net result is a fusion-incompetent SV that futilely occupies release sites. Structures correspond to the key in Figure 2.

**Figure 6:**
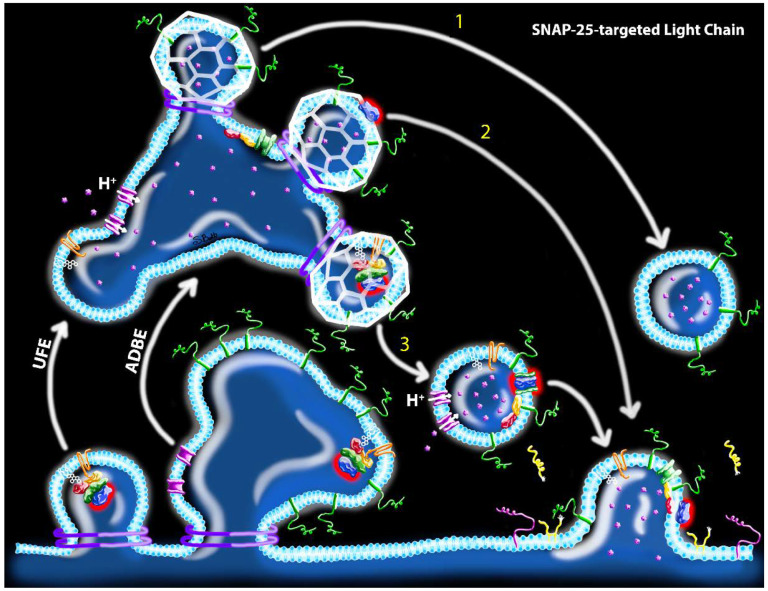
Hypothesized mechanisms of toxin uptake and potential proteolytic sites of action of SNAP-25-targeted LCs following UFE or ABDE. After fission from the presynaptic membrane, ABDE produces a bulk endosome, whereas UFE produces an endosome vesicle that subsequently fuses with a bulk endosome. vATPase proton pumps are activated during the bulk endosome stage, leading to slow acidification of bulk endosomes. Nascent SVs are produced from bulk endosomes via clathrin-coated (white) biogenesis with three potential outcomes: (1) sLC (blue with red halo) translocation occurs in bulk endosome, producing SVs that do not carry sLC; (2) Similar to step 1, except a translocated sLC is captured on the new SV; and (3) Acidification of the bulk endosome is not sufficient to drive translocation and intact holotoxin is captured within the SV during biogenesis. sLC translocation occurs after clathrin uncaging, producing SVs with active sLC. All scenarios produce fusion-competent SVs; however, in scenarios 2 and 3, sLC is deposited on the presynaptic membrane, where it subsequently cleaves SNAP-25 (yellow) in the vicinity of release sites. Structures correspond to the key in Figure 2.

However, the CME model raises questions as to whether stoichiometric inhibition of SVs by vLC is sufficient to cause paralysis. Although mammalian NMJs contain approximately 10^3^ docked vesicles in the readily releasable pool [66,67,68], it is estimated that fewer than 100 LC/B molecules are sufficient to fully paralyze a mouse phrenic motor endplate [c.f. 20]. Thus, it is difficult to reconcile how a relatively small number of vLC molecules could block release at large numbers of release sites, particularly if vLCs remain associated with a single SV. While this disjunction could be reconciled if vLC moved among SVs, the corresponding mechanism is unclear unless the toxin remains cytosolic as discussed above. A more plausible answer would be if LC translocation and VAMP cleavage occurred on bulk endosomes prior to SV biogenesis. Indeed, the acidification of bulk endosomes is necessary for SV generation during UFE and ADBE, thus producing the molecular conditions necessary for translocation to occur [56,57,69]. Consistent with this hypothesis, ultrastructure studies reveal association of radiolabeled BoNT with presynaptic endosomes [65,70,71]. According to this hypothesis, translocated vLC could cleave VAMP proteins on the synaptic endosome membrane, producing SVs that were fusion-incompetent yet not associated with a vLC molecule (Figure 5). Similarly, sLC could cleave endosomal SNAP-25 proteins internalized as *cis*-SNARE complexes during endocytosis [72], contributing to the local pool of cleaved SNAP-25 on the presynaptic membrane following a fusion event (Figure 6). Moreover, sLC and vLC would continue to proteolyze endosomal SNARE proteins until they were (1) degraded or cleared directly from the endosome or (2) become captured on a newly budded SV, at which time they will replicate the CME model describe above. It is tempting to further speculate that vLC and sLC evolved distinctive strategies to optimize intoxication efficiency, i.e., molecular interactions that stabilize vLC activity at the endosome while sLC undergoes rapid trafficking to the presynaptic membrane. However, comparative studies of intracellular trafficking and protein-toxin interactions have yet to be done.

### 2.3. Mode of Endocytosis May Influence the Rate of Neuronal Intoxication

In addition to the location of translocation and the stoichiometry of BoNT LC to SVs, the mode of synaptic endocytosis may affect the latency between intoxication and depression of neuromuscular transmission. SVs are broadly classified into two populations based on functional characteristics: the readily releasable pool (RRP), which comprises the small percentage (~1%) of vesicles that are docked at the active zone and available to undergo fusion, and the reserve pool, which replenishes the RRP during periods of intense neuronal activity [3]. The reserve pool is further stratified into subpopulations that are progressively recruited under conditions of increasing demand. Not only do the different modes of endocytosis have different rates of SV biogenesis, but they also target regenerated SVs to distinct pools, raising the possibility that the dominant mode of synaptic endocytosis may affect the timing of neuromuscular paralysis by altering toxin trafficking and localization. Indeed, the RRP is selectively replenished by CME-derived SVs, whereas ADBE-derived SVs are targeted to the reserve pool [45]. Thus, depending on the rate of exocytosis, delivery of intoxicated SVs (meaning SVs associated with LC or impaired by LC) to the active zone membrane may require multiple rounds of evoked release before they enter the RRP and fuse to deposit sLC on active zone membrane, or alternatively futilely occupy release sites due to the vLC cleavage of VAMP.

### 2.4. Does Mode of Endocytosis Affect the Amount of Toxin Uptake?

A clear relationship between synaptic transmission and BoNT intoxication has been demonstrated clinically by more severe paralysis in response to nerve stimulation during intoxication [70,73] and in cultured neurons by greater SNARE protein cleavage following intoxication under depolarizing conditions [74]. This has been interpreted to indicate that nerve stimulation concomitantly increases presynaptic BoNT load through greater presynaptic availability of BoNT receptors due to additional rounds of vesicle fusion. Mammalian motor neurons exhibit highly variable firing rates [75], and thus individual nerve terminals may engage in multiple modes of synaptic endocytosis, raising the question of whether the mode of endocytosis affects the quantity of toxin uptake. Regardless of the specific mode of endocytosis, motor nerve terminals must retrieve quantities of membrane and SV protein–including BoNT receptors–that correlate to the amount of fused SV material to avoid destabilizing neurological function. However, physicochemical constraints specific to each mode of endocytosis may influence toxin uptake, such as endosomal vesicle size and composition. Indeed, we have already described how pore size during kiss-and-run endocytosis is unlikely to allow BoNT uptake. Alternatively, could the size of endocytosed vesicles present an upper constraint on toxin load? A 40 nm diameter CME vesicle (the smallest endosomal vesicle) has an aqueous volume of 12,696 nm^3^, which is theoretically sufficient to accommodate up to 24 toxin molecules [76,77]. In contrast, UFE or ADBE produce endosomes that are 50-fold to 200-fold larger [33,78], and thus endosomal vesicles could accommodate greater numbers of toxin molecules. However, CME vesicles contain 2–5 copies of SV2 and 5–7 copies of SYT1 [24,77,79,80], thus limiting toxin uptake to a maximum of 5–7 BoNT molecules per CME vesicle. Because the primary limiting factor in toxin uptake among CME, ABDE and UFE appears to be the number of BoNT receptors, which is a fixed ratio of the number of fusion events, it appears that the mode of endocytosis has minimal effect on presynaptic BoNT load. Alternatively, as described above, the principal physiological difference among endocytotic modes is likely to be the location of LC activation and latency to impairment of SV fusion.

## 3. Conclusions

In this review, we discuss potential implications of newly discovered modes of synaptic vesicle recycling on BoNT uptake and LC trafficking. These findings provide potential solutions to persistent questions regarding the pharmacology of intracellular LC trafficking. In addition to hypothesizing novel mechanisms of toxin activation, we introduce the concept that molecular toxicity may manifest upstream of SV generation. A combination of functional, biochemical, ultrastructural and single-molecule tracking studies will be required to experimentally determine the mode(s) of synaptic endocytosis involved in BoNT uptake, the location(s) of LC translocation and where and when SNARE protein cleavage occurs. Clarifying the mechanistic relationship between molecular toxicity and impaired SV fusion is important for understanding the neuron-toxin interactions responsible for toxic manifestations as well as for designing broad-spectrum therapeutics that exploit specific mechanisms of toxin trafficking to block or inhibit intracellular LC activity.

## Abbreviations

BoNT	botulinum neurotoxin
SNARE	soluble N-ethylmaleimide-sensitive fusion protein attachment protein receptors
HC	heavy chain
LC	light chain
SV	synaptic vesicle
CME	clathrin-mediated endocytosis
UFE	ultrafast endocytosis
ADBE	activity-dependent bulk endocytosis
vLC	VAMP1-3-targeted light chain
sLC	SNAP-25-targeted light chain
RRP	readily releasable pool
